# Intracellular rupture, exocytosis and actin interaction of endocytic vacuoles in pancreatic acinar cells: initiating events in acute pancreatitis

**DOI:** 10.1113/JP275879

**Published:** 2018-06-06

**Authors:** Michael Chvanov, Francesca De Faveri, Danielle Moore, Mark W. Sherwood, Muhammad Awais, Svetlana Voronina, Robert Sutton, David N. Criddle, Lee Haynes, Alexei V. Tepikin

**Affiliations:** ^1^ Department of Cellular and Molecular Physiology and NIHR Liverpool Pancreas Biomedical Research Unit University of Liverpool Liverpool UK

**Keywords:** actin, acute pancreatitis, endocytic vacuoles, endocytosis, exocytosis, pancreas, pancreatic acinar cells, trypsin

## Abstract

**Key points:**

- Giant trypsin‐containing endocytic vacuoles are formed in pancreatic acinar cells stimulated with inducers of acute pancreatitis.
- F‐actin envelops endocytic vacuoles and regulates their properties.
- Endocytic vacuoles can rupture and release their content into the cytosol of acinar cells.
- Endocytic vacuoles can fuse with the plasma membrane of acinar cells and exocytose their content.

**Abstract:**

Intrapancreatic activation of trypsinogen is an early event in and hallmark of the development of acute pancreatitis. Endocytic vacuoles, which form by disconnection and transport of large post‐exocytic structures, are the only resolvable sites of the trypsin activity in live pancreatic acinar cells. In the present study, we characterized the dynamics of endocytic vacuole formation induced by physiological and pathophysiological stimuli and visualized a prominent actin coat that completely or partially surrounded endocytic vacuoles. An inducer of acute pancreatitis taurolithocholic acid 3‐sulphate and supramaximal concentrations of cholecystokinin triggered the formation of giant (more than 2.5 μm in diameter) endocytic vacuoles. We discovered and characterized the intracellular rupture of endocytic vacuoles and the fusion of endocytic vacuoles with basal and apical regions of the plasma membrane. Experiments with specific protease inhibitors suggest that the rupture of endocytic vacuoles is probably not induced by trypsin or cathepsin B. Perivacuolar filamentous actin (observed on the surface of ∼30% of endocytic vacuoles) may play a stabilizing role by preventing rupture of the vacuoles and fusion of the vacuoles with the plasma membrane. The rupture and fusion of endocytic vacuoles allow trypsin to escape the confinement of a membrane‐limited organelle, gain access to intracellular and extracellular targets, and initiate autodigestion of the pancreas, comprising a crucial pathophysiological event.

## Key points

- Giant trypsin‐containing endocytic vacuoles are formed in pancreatic acinar cells stimulated with inducers of acute pancreatitis.
- F‐actin envelops endocytic vacuoles and regulates their properties.
- Endocytic vacuoles can rupture and release their content into the cytosol of acinar cells.
- Endocytic vacuoles can fuse with the plasma membrane of acinar cells and exocytose their content.

## Introduction

Pancreatic acinar cells secrete digestive enzymes and precursors of digestive enzymes (zymogens) by stimulated exocytosis (Williams, 2008; Yule, 2010; Thorn & Gaisano, 2012). In the acinar cells, zymogens are packaged into large secretory granules (also termed zymogen granules). Exocytosis induced by physiologically‐relevant secretagogues cholecystokinin and acetylcholine in this cell type is mediated by Ca^2+^ signalling cascades (Petersen & Tepikin, 2008; Williams, 2008; Thorn & Gaisano, 2012; Liang *et al*. 2017). Ca^2+^‐dependent secretion of zymogens involves compound exocytosis in which secretory granules undergo fusion not only with the plasma membrane, but also with each other, forming large post‐exocytic structures (Nemoto *et al*. 2004; Sherwood *et al*. 2007; Thorn & Gaisano, 2012), which can disconnect from the plasma membrane forming endocytic vacuoles (EVs). Under physiological conditions, exocytosis of zymogen granules is limited to the apical region of the cell; however, under pathophysiological conditions, basolateral exocytosis can also occur (Cosen‐Binker *et al*. 2008). Actin filaments play an important role in regulating exocytosis in pancreatic acinar cells (Muallem *et al*. 1995; Valentijn *et al*. 2000; Nemoto *et al*. 2004; Larina *et al*. 2007; Jang *et al*. 2012). In particular, actin has been shown to interact with post‐exocytic structures (Jang *et al*. 2012). Following exocytosis, zymogens secreted by the acinar cells are transported via the system of pancreatic ducts into the duodenum. This transport relies on fluid secretion by pancreatic acinar and pancreatic ductal cells (Hegyi & Rakonczay, 2015; Pallagi *et al*. 2015). Under physiological conditions, activation of zymogens occurs specifically in the intestine. However, during acute pancreatitis, some of the zymogens become activated inside the pancreas itself rather than the intestine (Leach *et al*. 1991; Hofbauer *et al*. 1998) initiating pancreatic autodigestion. Trypsin is the key digestive enzyme involved in this process; intrapancreatic trypsinogen activation (i.e. formation of trypsin) is an early step in the development of acute pancreatitis and is observed in both the clinical setting and experimental models of this disease (Gudgeon *et al*. 1990; Hofbauer *et al*. 1998; Otani *et al*. 1998; Halangk *et al*. 2000; Van Acker *et al*. 2007; Dawra *et al*. 2011; Pallagi *et al*. 2011; Sendler *et al*. 2018). The mechanism of trypsinogen activation in the pancreas is debated, with a considerable body of evidence suggesting the involvement of cathepsin B in this process (Saluja *et al*. 1997; Halangk *et al*. 2000; Lerch & Halangk, 2006; Sendler *et al*. 2018). Trypsinogen activation in pancreatic acinar cells is an important contributor to the damage and death of the acinar cells *in vitro* and the damage of pancreatic tissue in *in vivo* models (Ji *et al*. 2009; Kereszturi & Sahin‐Toth, 2009; Mareninova *et al*. 2009; Dawra *et al*. 2011; Gaiser *et al*. 2011). Intracellular activation of trypsinogen has been described (Leach *et al*. 1991; Hofbauer *et al*. 1998; Otani *et al*. 1998; Kruger *et al*. 2000; Raraty *et al*. 2000); however, the nature of the initiating organelle(s) is still debated. Functioning vacuolar ATPase (V‐ATPase) and acidic intraorganellar milieu are required for zymogen activation (Lerch *et al*. 1993; Waterford *et al*. 2005; Kolodecik *et al*. 2009). Vacuolization is a hallmark of the acinar cell damage triggered by inducers of acute pancreatitis (Watanabe *et al*. 1984; Hofbauer *et al*. 1998; Otani *et al*. 1998; Sherwood *et al*. 2007; Mareninova *et al*. 2009; Kim *et al*. 2011). Both endocytic and non‐EVs have been described (Voronina *et al*. 2007). In our previous study, we observed trypsinogen activation in EVs (Sherwood *et al*. 2007) and this prompted us to further investigate these cellular structures.

The initial aim of the present study was to characterize the dynamics of the formation of EVs under physiological and pathological conditions. However, early in the project, we serendipitously discovered three new phenomena: rupture, exocytosis and actination of the EVs. The project consequently refocused on characterizing these novel cellular events.

## Methods

### Ethical approval and laboratory animals

Pancreata were obtained from adult (6–8 weeks old) male CD1mice (Charles River, Margate, Kent, UK). The animals were humanely killed by cervical dislocation (schedule 1 procedure) in accordance with the Animals (Scientific Procedures) Act (1986) under Establishment Licence 40/2408 and with approval by the University of Liverpool Animal Welfare Committee. Prior to experiments, mice had *ad libitum* access to food and water.

### Chemicals

Lucifer yellow (LY) and BZiPAR (fluorogenic probe for trypsin activity) (Kruger *et al*. 1998; Kruger *et al*. 2000; Raraty *et al*. 2000; Sherwood *et al*. 2007) were obtained from Thermo Fisher Scientific (Waltham, MA, USA). Fluorescein isothiocyanate–dextran MW 40,000 (FITCD) was obtained from Sigma‐Aldrich (St Louis, MO, USA). Dextran Texas Red 3000 MW neutral (TRD) and other fluorescence‐labelled dextrans obtained were from Thermo Fisher Scientific. Collagenase was obtained from Worthington Biochemical (Lorne Laboratories, Lower Earley, UK). Thapsigargin was obtained from Calbiochem (San Diego, CA, USA). The SiR‐Actin kit was obtained from Spirochrome (Stein am Rhein, Switzerland). CA‐074 and bafilomycin A1 were obtained from Tocris Bioscience (Bristol, UK) and CA‐074Me was obtained from Calbiochem. Disulpho‐cyanine5 carboxylic diS‐cy5 acid (diS‐Cy5) was obtained from Cyandye LLC (Sunny Isles Beach, FL, USA). CBQCA Atto‐tag was obtained from Setarech Biotech (Eugene, OR, USA) and the rest of the chemicals, including DMSO, benzamidine, cholecystokinin (CCK) and taurolithocholic acid 3‐sulphate (TLC‐S), were obtained from Sigma‐Aldrich.

### Solutions

A standard extracellular solution used for cell isolation and measurements of vacuole formation contained (in mm): 140 NaCl, 4.7 KCl, 1.13 MgCl_2_, 1 CaCl_2_, 10 d‐glucose and 10 Hepes. The solution was adjusted to pH 7.3 using NaOH. CCK and TLC‐S were added to the standard extracellular solution to attain the concentrations specified in the description of individual experiments.

### Cell preparation, labelling and imaging

The pancreatic acinar cells were isolated by digestion with purified collagenase (200 units mL^–1^) as described previously (Chvanov *et al*. 2015). Freshly isolated pancreatic acinar cells were dispersed in standard extracellular solution, plated on to poly‐l‐lysine‐coated glass‐bottomed Petri dishes from MatTek (Ashland, MA, USA) and maintained at 35°C. Extracellular solution was then removed and replaced with the incubation solution containing fluorescence probe(s) (e.g. LY or TRD) plus test compound (e.g. CCK) as specified in the description of the individual experiments. In some experiments, we utilized small pancreatic sections (∼1 mm in liner dimensions), which were produced by cutting pancreas with a surgical scalpel blade. The sections were then handled and labelled using procedures that were the same as those for the isolated cells. Only surface cells of the sections were imaged and analysed.

Images of cells and organelles were obtained using a confocal laser scanning microscope (TCS SL or a TCS SP2; Leica Microsystems, Wetzlar, Germany); the axial resolution in our experiments was ∼1 μm. Fluorescence of LY was excited using a 458 nm laser line; emission was recorded at wavelengths 481–572 nm. Fluorescence of diS‐Cy5 was excited using a 633 nm laser line; emission was recorded at wavelengths 650–700 nm. Fluorescence of Texas Red‐labelled dextrans (TRDs) was excited using 543 nm laser line; emission was recorded at wavelengths 595–703 nm. Fluorescence of Tetramethylrhodamine‐labelled dextrans (TMRDs) was excited using a 543 nm laser line; emission was recorded at wavelengths 560–680 nm. Fluorescence of FITCD was excited using a 488 nm laser line; emission was recorded at wavelengths 505–530 nm. In our experiments, the fluorescence‐labelled dextrans were used at concentrations that correspond to ∼100 μm of the labelled fluorescent molecules (the number of fluorescent molecules associated with each molecule of dextran is different for dextrans of different molecular weight and can also vary between the batches of the labelled dextrans).

The procedure used for labelling trypsinogen comprised: 100 mg of bovine trypsinogen and 8.2 mg of *N*‐acetyl‐cysteine (NAC) dissolved in 5 mL of aqueous solution containing 2 mm of Hepes (pH 11.0 adjusted with NaOH). Then, 10 mg of CBQCA Atto‐tag was dissolved in the mixture containing 180 μL of DMSO and 120 μL of ethanol and slowly added dropwise to the stirred trypsinogen‐NAC mixture. After 15 min, the pH of the solution was adjusted to 7.0 with HCl and each 2.5 mL of the reaction mixture was eluted from a pre‐equilibrated PD‐10 desalting column (Amersham Biosciences) using 3.5 mL of normal extracellular buffer, in accordance with the manufacturer's instructions. This solution was ready to be used in experiments with live cells (or could be frozen for storage). Fluorescence of CBQCA Atto‐trypsinogen was excited using a 488 nm laser line; emission was recorded at wavelengths 580–630 nm.

In experiments testing the accumulation of fluorescence probes with different molecular weights in EVs, cells were maintained in suspension containing the combinations of probes (LY and a fluorescence‐labelled dextran, or diS‐Cy5 and a fluorescence‐labelled trypsinogen) plus CCK (10 pm or 10 nm) for 30 min at 35°C. Cells were then placed in glass‐bottomed Petri dish from MatTek, allowed to adhere, and then washed three times with standard extracellular solution to remove CCK and endocytic tracers. The cell‐containing MatTek dish was then moved to the stage of an inverted confocal microscope for imaging. During the imaging, cells were maintained in extracellular solution at 35°C.

In experiments involving simultaneous imaging of EVs and trypsin activity, the cells were immersed in a solution containing 100 μm of dextran Texas Red 3000 MW neutral and 100 μm BZiPAR. BZiPAR is a fluorogenic probe for trypsin activity; upon cleavage by trypsin, it is first converted to fluorescent monoamine, which is further cleaved by trypsin and releases a fluorescent molecule R110 (Leytus *et al*. 1983) (https://tools.thermofisher.com/content/sfs/manuals/mp06501.pdf). Fluorescence of BZiPAR products was excited using a 488 nm laser line; emission was recorded at wavelengths 500–530 nm.

To quantify the number of cells with ruptured vacuoles using increased cytosolic fluorescence, the cells were incubated in the presence 100 μm of diS‐Cy5 (and in some cases 100 μm of Texas Red dextran) for 2 h at 35°C. Specified agonists and/or inhibitors were added to the incubation media. The cells were then washed by perfusion to remove the fluorescence probes from the extracellular solution. After washing, fluorescence images of the cells were acquired and analysed by drawing the region of interest that included the cytosol of each individual cell but excluded all stained vacuoles and plasma membrane (i.e. the cell boundary resolvable on images). Next, the data concerning cytosolic fluorescence collected using this procedure were pooled together from all experimental groups (typically comprising 100–300 data points) to obtain a frequency distribution plot. This frequency histogram was then approximated by Gaussian best fit (multipeak). The first peak (the one closer to zero) was interpreted as the one representing the cells, in which there was no increase of cytosolic fluorescence as a result of EV rupture. After this, the threshold of the central value (corresponding to the maximal cell number) plus three half‐widths (three‐sigma) of the first peak was calculated, and the cells whose cytosolic fluorescence was above the threshold were considered to demonstrate EV rupture leading to a cytosolic rise of fluorescence. The proportion of cells with increased cytosolic fluorescence was calculated for each specified experimental condition and compared with the appropriate control. If the first two peaks of the approximation did not separate (the value corresponding to the maximal amplitude plus the half‐width of the first peak were more than the central value for the second peak), then such an experiment was excluded from further analysis.

To image F‐actin distribution, cells were infected with adenovirus containing LifeAct construct (ibidi GmbH, Martinsried, Germany) by adding adenovirus (10^7^ pfu mL^–1^) into a dish containing acinar cells and incubating for 10–12 h at 35°C. Before performing experiments, the adenovirus was washed off with extracellular solution. Cell expressing LifeAct were imaged on an LSM 710 (Carl Zeiss, Oberkochen, Germany) at 35°C with excitation using a 543 nm laser line; emission was recorded at wavelengths 560–600 nm. Another approach for F‐actin labelling involved SiR‐actin. To reveal F‐actin distribution using this probe, cells were incubated in solution containing 1 μm SiR‐actin and 10 μm verapamil for 1 h at 34.5°C prior to stimulation with supramaximal CCK. In these experiments, cells were fixed after CCK stimulation by incubation with 1.8% paraformaldehyde for 48 h at +4°C, washed with PBS and imaged on a TCS SL (Leica) with excitation using a 633 nm laser line; emission was recorded at wavelengths 650–795 nm.

The results are reported as the mean ± SEM. Statistical comparisons were performed using Student's *t* test or ANOVA followed by Dunnet's test. *P* < 0.05 was considered statistically significant.

## Results

### Labelling, formation and sizes of EVs

**Figure 1:**
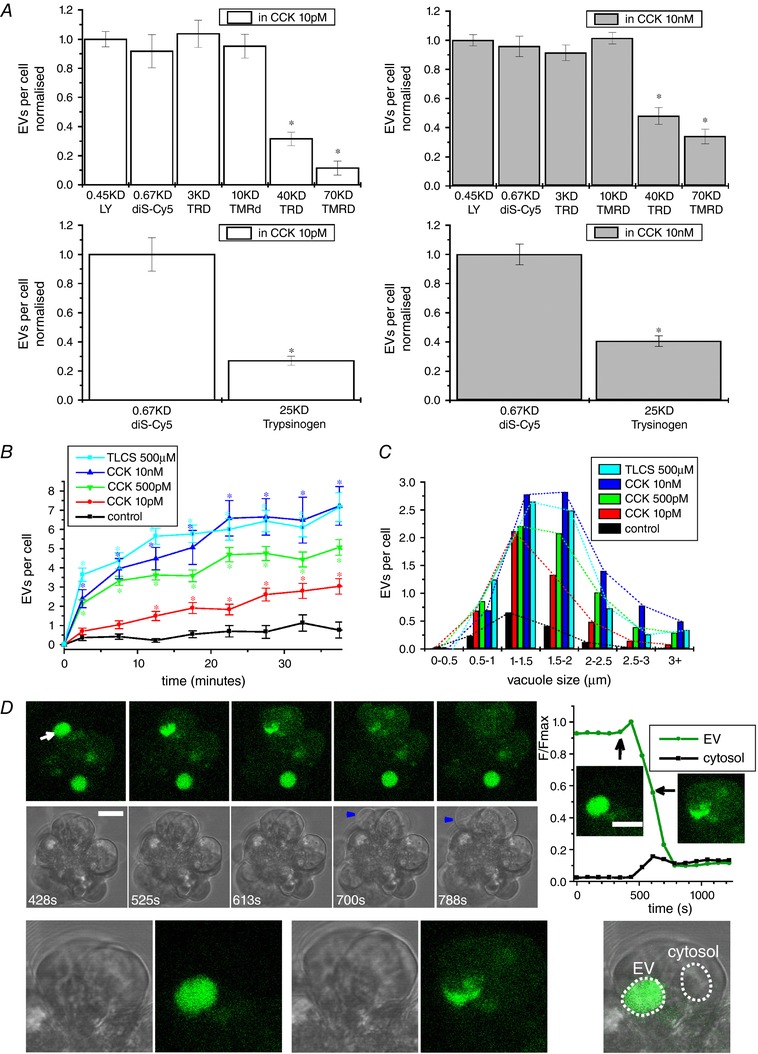
Labelling, formation and rupture of EVs *A*, upper: showing the ability of molecules with different molecular weights to enter and label EVs in cells stimulated by physiological (left) and supramaximal (right) CCK concentrations. The EVs were revealed by accumulation of low molecular weight probe LY. In these experiments, we also used fluorescence‐labelled dextrans with different molecular weights: dextran Texas Red 3000 MW neutral (TRD 3 kDa; this smallest of the labelled dextrans is referred to as TRD in the remainder of the text; here, we also specify its molecular weight to distinguish it from the larger fluorescence‐labelled dextrans), dextran tetramethylrhodamine 10,000 MW neutral (TMRD 10 kDa), dextran Texas Red 40,000 MW neutral (TRD 40 kDa) and dextran tetramethylrhodamine 70,000 MW neutral (TMRD 70 kDa). The ratios of the number of vacuoles containing labelled dextrans to the total number of vacuoles revealed by LY are shown; *statistically significant difference from the total number of the vacuoles (P < 0.05). Lower: the ratios of the number of EVs containing labelled bovine trypsinogen (CBQCA Atto‐trypsinogen) to the total number of vacuoles (revealed by diS‐Cy5) in cells stimulated with 10 pm CCK and 10 nm CCK. The proportion of vacuoles stained with CBQCA Atto‐trypsinogen was significantly higher in cells stimulated with supramaximal CCK. *B*, time course of EV formation in unstimulated cells and cells stimulated with CCK or TLC‐S; *statistically significant difference (*P* < 0.05) from control (unstimulated cells). *C*, distribution of EV diameters in control (unstimulated) cells and cells stimulated for 30 min with CCK or TLC‐S. The mean ± SEM diameter was: 1.47 ± 0.04 μm (*n* = 190 vacuoles) under control conditions (unstimulated cells); 1.50 ± 0.03 μm (*n* = 384 vacuoles) for CCK 10 pm; 1.68 ± 0.04 μm (*n* = 264 vacuoles) for CCK 500 pm; 1.79 ± 0.04 μm (*n* = 403 vacuoles) for CCK 10 nm; and 1.62 ± 0.05 μm (*n* = 297 vacuoles) for TLCS 500 μm. The vacuoles induced by supramaximal CCK concentrations and TLC‐S were larger than the vacuoles induced by the physiological concentration of CCK (*P* < 0.05). *D*, rupture of an EV membrane and the loss of its content into cytosol in a cell stimulated by 10 nm CCK. The rupturing vacuole is indicated by the white arrow. Blue arrowheads indicate cell membrane blebs. The inset on the right shows the EV undergoing rupture in an expanded scale and the traces of fluorescence recorded from the EV and the cytosol (the regions of interest are shown by dashed lines); black arrows on the graph indicate the time points and fluorescence intensities corresponding to the inset images. Scale bar = 10 μm.

EVs are rapidly formed intracellular structures that can be labelled with membrane impermeant fluorescent dyes, including fluorescence‐labelled dextrans (Fig. 1). The numbers of vacuoles labelled by small molecules LY and diS‐Cy5 were similar to that labelled by fluorescent dextrans with molecular weights of 3 and 10 kDa (Fig. 1 *A*). Dextrans with molecular weights of 40 and 70 kDa were partially excluded from the EVs (Fig. 1 *A*), suggesting a relatively small diameter of the fusion pore. Similar partial exclusion was observed for the fluorescence‐labelled trypsinogen (Fig. 1 *A*). Fluorescence probes LY, diS‐Cy5 and TRD produced similar labelling of EVs and were used for detecting and investigating the properties of EVs in the present study.

Supramaximal concentrations of CCK (500 pm and 10 nm) and toxic bile acid TLC‐S (500 μm) induced vigorous vacuolization (Fig. 1 *B*). The number and, importantly, the size of the EVs induced by supramaximal CCK or TLC‐S was substantially larger than that induced by the physiological concentration of CCK (10 pm) or observed in unstimulated cells (Fig. 1 *B* and *C*). Notably, EVs larger than 2.5 μm were almost exclusively observed in cells treated with supramaximal CCK or TLC‐S (Fig. 1 *C*).

### Rupture and actin interaction of EVs

The large size of the EVs, formed as a result of pathologically relevant stimulation, allowed us to reveal the rupture of these organelles. Upon cell stimulation with supramaximal doses of CCK, some of the EVs ruptured inside the cells so that the probe escaped from the EVs into the cytosol and increased cytosolic fluorescence (*n* = 30) (Fig. 1 *D*). The observation time necessary to resolve one rupture event was ∼8 h (∼480 min). To resolve 30 ruptures, we imaged/analysed 220 vacuole‐containing cells. The loss of the indicator from an EV could be either complete (*n* = 17) (Fig. 1 *D*) or partial (*n* = 13) (not shown). In the majority of experiments, EV rupture was followed by blebbing of the plasma membrane (Fig. 1 *D*) and cell death, suggesting that EV rupture is cytotoxic. Cell death occurred 27 ± 4 min after the rupture (*n* = 30). In these experiments, we have studied vacuoles with a diameter greater than 2.5 μm. The large size of the EVs undergoing rupture is essential for the positive identification of such events. The increase of cytosolic fluorescence, detectable during rupture of a large EV, strongly indicates the classification of the event as a rupture. In this respect, the large size of EVs formed by stimulation with supramaximal CCK or TLC‐S is particularly important.

**Figure 2:**
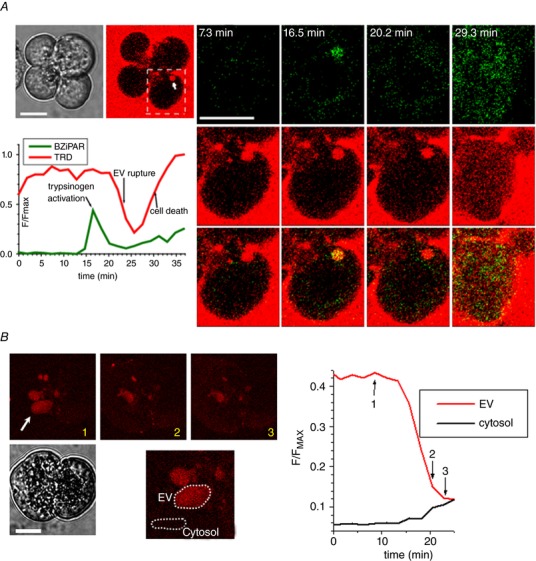
Activation of trypsinogen and rupture of an EV *A*, cells were stimulated with 10 nm CCK. EVs were revealed by accumulated dextran Texas Red 3000 MW neutral (TRD). Left: cluster of acinar cells (transmitted light and TRD fluorescence). The EV containing TRD is indicated by a white arrow. Scale bars = 10 μm. The boundary of the region shown in the right gallery of images is indicated by the white dashed line. Trypsinogen activation (i.e. increase of fluorescence as a result of BZiPAR cleavage) is shown in green. Note the trypsinogen activation in the EV that started after the vacuole was formed. The intravacuolar increase of BZiPAR‐mediated fluorescence was usually transient; the decline of fluorescence started before the rupture of the vacuole (i.e. before the loss of TRD). A possible reason for this is that the final product of BZiPAR cleavage R110 is a membrane permeant probe and therefore poorly retained in cellular compartments. Changes of fluorescence recorded in the region of interest containing the vacuole are shown. At the end of the experiment, we observed an increase of TRD fluorescence in the cytosol of the cell, signifying the loss of the plasma membrane integrity (i.e. cell death). Right column: the beginning of this process. *B*, Lower left: doublet of pancreatic acinar cells in transmitted light. Scale bar = 10 μm. EVs were revealed by accumulated TRD. In this type of experiment, benzamidine (1 mm) was applied at least 30 min before the start of experiment and maintained in the extracellular solution for the duration of the experiment. Cells were stimulated by 10 nm CCK. The rupturing EV is indicated by the white arrow. The insert on the lower left shows the EV undergoing rupture on an expanded scale. The traces of fluorescence shown on the right were recorded from the EV (red) and the cytosol (black); the specific regions of interest are indicated by dashed lines on the lower left panel. The numbers on the traces (1–3) correspond to the numbers on the images and define the time points at which the images and corresponding fluorescence intensities were recorded.

EVs are the sites of trypsinogen activation in pancreatic acinar cells. Using combined application of TRD (to detect EVs) and BZiPAR (a fluorogenic probe for trypsin activity), we observed trypsinogen activation occurring prior to the rupture of an EV (*n* = 13) (Fig. 2 *A*). Notably, we have observed vacuole formation and rupture in CCK‐stimulated cells incubated with the trypsin inhibitor benzamidine (*n* = 13) (Fig. 2 *B*). Importantly, 1 mm benzamidine used in these experiments, eliminates resolvable trypsinogen activation in pancreatic acinar cells (Sherwood *et al*. 2007). These experiments suggest that trypsin is not critically important for the rupture of EVs.

Similar results were obtained in experiments with the cathepsin B inhibitor CA074, which was designed by Towatari *et al*. (1991) to specifically inhibit this protease. In these experiments, combination of CA074 (10 μm) with a more cell‐permeable methyl ester form CA074‐Me (1 μm) was applied at least 30 min before the experiment and maintained in the extracellular solution for the duration of the experiment. This combination of CA074 and CA074‐Me is abbreviated as CA074/Me in the present study. In the cells stimulated by 10 nm CCK, CA074/Me did not prevent rupture of EVs (*n* = 8) (not shown), suggesting that cathepsin B is probably not responsible for the rupture.

**Figure 3:**
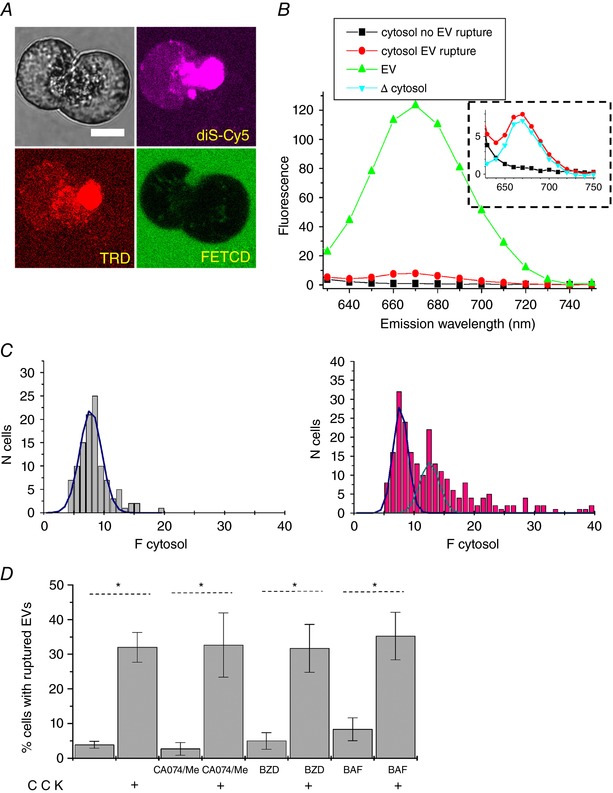
Visualizing the appearance of membrane‐impermeant fluorescence probes in the cellular cytosol *A*, cells were stimulated by 10 nm CCK for 2 h at 35 °C in the presence of diS‐Cy5 and dextran Texas Red 3000 MW neutral (TRD). Cells were then washed by perfusion with standard extracellular solution. Following the wash, cells were immersed in the extracellular solution containing FITCD and imaged. Upper left: transmitted light image of the cells. Scale bar = 10 μm. Images of the fluorescence of the cells and extracellular milieu recorded using excitation/emission corresponding to the specified probes shown elsewhere. Note the EV in the right cell and increased cytosolic fluorescence on diS‐Cy5 and TRD images of the left cell. The FITCD image indicates that the plasma membrane of the cell is intact. *B*, representative fluorescence emission spectra recorded from cells stimulated as in (*A*) but in the presence of diS‐Cy5 only. The fluorescence was excited by a 595 nm laser line. The graphs show fluorescence spectra recorded from an EV (green), cytosol of a cell that satisfies criteria for detecting the EV's rupture/leakage (red; see Methods and *C*), cytosol of a cell that did not satisfy criteria for detecting rupture/leakage (black; see Methods and *C*). The blue trace in the insert is produced by subtracting black trace (mainly determined by cellular autofluorescence) from the cytosolic fluorescence of a cell that satisfies the criteria for detecting rupture/leakage. The residual trace shows a spectrum that is similar to the spectrum of diS‐Cy5 recorded from an EV. The red and black traces were recorded from different cells and shown on the same graph for illustration. *C*, left: intensities of cytosolic fluorescence in cells that were immersed in the indicator‐containing diS‐Cy5 solution for 30 min but were not stimulated. Only a very few small vacuoles are expected to form during this period of time (Fig. 1 *B* and *C*) and the distribution should therefore reflect cytosolic fluorescence in the cells that did not have ruptured EVs. The blue trace represents a single Gaussian approximation of the distribution. Right: frequency histogram of cells after two hours of incubation with diS‐Cy5. The CCK concentration was 10 nm. The first two Gaussian peaks of the approximation are shown by blue and magenta lines. Cells with cytosolic fluorescence above threshold (central value of the first peak plus 3 sigma) are classified as the cells that experienced rupture/leakage of EV(s). *D*, the method illustrated in (*A*) to (*C*) was used to evaluate the proportions of cells with ruptured vacuoles. CCK concentration was 10 nm (in specified experiments). Neither inhibition of serine protease with benzamidine (1 mm), nor inhibition of cathepsin B with combination of CA074 (10 μm) and CA074‐Me (1 μm) (abbreviated as CA074/Me) produced a significant difference in the proportion of cells with increased cytosolic fluorescence from control. Inhibition of V‐ATPase with 100 nm of bafilomycin A1 (Baf) also did not produce a statistically significant change in the proportion of cells with increased cytosolic fluorescence. The number of experiments in each condition was: *n* = 20 experiments for control (unstimulated cells) and CCK; *n* = 9 for CA074/Me and CA074/Me + CCK; *n* = 8 for benzamidine and benzamidine + CCK; *n* = 6 for bafilomycin A1 and bafilomycin A1 + CCK. Each of the individual experiments involved acquisition and analysis of a fluorescence distribution similar to that shown on the right of (*C*).

A considerable confocal observation time (∼8 h, comprising two or three confocal sessions) was required to resolve a single rupture of an EV. These direct imaging experiments provided a clear demonstration of the phenomenon, although they were challenging, time consuming and not conducive to an investigation of the processes modulating the ruptures. We therefore developed and utilized a complementary technical approach for investigating possible roles of trypsin and cathepsin B in the rupture of the vacuoles. This approach relies on the fact that a rupture of an EV containing fluorescence probe results in an increase of cytosolic fluorescence (Fig. 1 *D*). We have indeed observed a proportion of cells that had an intact plasma membrane but slightly increased cytosolic fluorescence with spectral properties corresponding to those of the probes utilized in our experiments studying EVs (diS‐Cy5 and/or TRD) (Fig. 3 *A* and *B*). Importantly, the proportion of such cells increased upon CCK stimulation (Fig. 3 *C* and *D*). Based on these observations, we inferred that the increase of cytosolic fluorescence is a result of the rupture or leakage of EVs. In these experiments, neither inhibition of trypsin (with 1 mm of benzamidine), nor inhibition of cathepsin B (with CA‐074/Me) produced statistically significant changes in the proportion of cells with the increased cytosolic fluorescence of diS‐Cy5 (Fig. 3 *D*). Inhibition of V‐ATPase is another mechanism for supressing intracellular/intraorganellar zymogen activation (Waterford *et al*. 2005; Kolodecik *et al*. 2009), including trypsinogen activation in EVs (Sherwood *et al*. 2007). In our experiments, 100 nm of bafilomycin A1, applied 30 min before CCK stimulation and maintained in the extracellular solution for the duration of the experiment, had no resolvable effect on the proportion of CCK‐stimulated cells with the increased cytosolic fluorescence of diS‐Cy5 (Fig. 3 *D*). Taken together, these observations support the notion that trypsin and cathepsin B are probably not involved in rupturing EVs.

**Figure 4:**
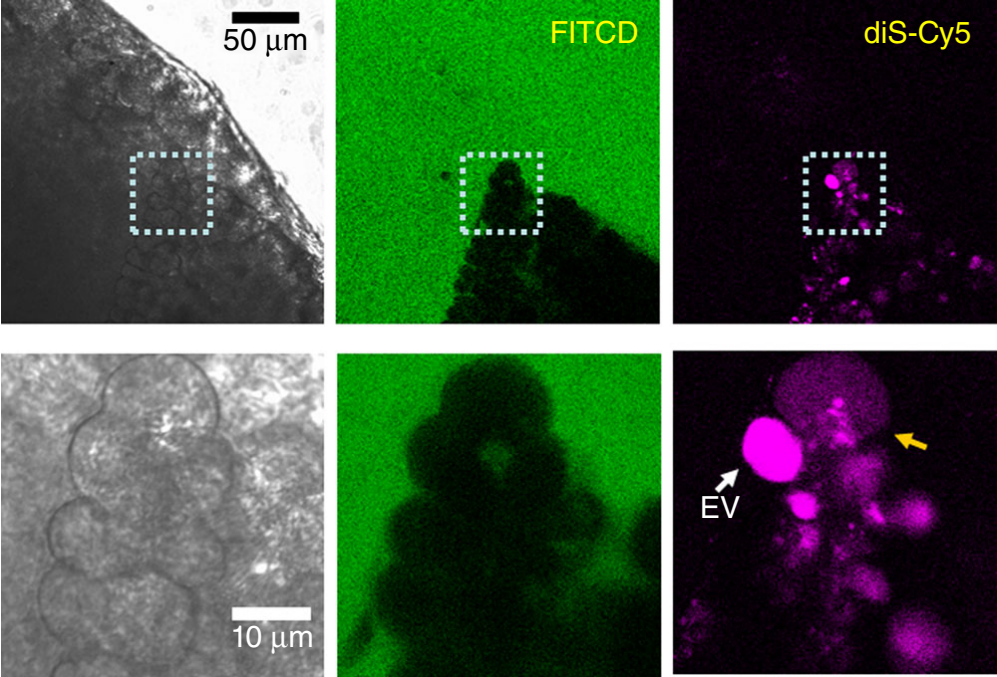
Cytosolic presence of membrane‐impermeant fluorescence probe in the cell located in undissociated pancreatic fragment Small (∼1 mm) section of mouse pancreas was stimulated by 100 nm CCK for 2 h at 35°C in the presence of diS‐Cy5 (shown in magenta), washed and imaged in the presence of FITCD (shown in green). The lower gallery of images depicts the fragment containing two cells within the section: one with a large intact EV (white arrow) and the adjacent cell with increased cytosolic fluorescence of diS‐Cy5. The FITCD image indicates that the plasma membrane of this cell is intact, suggesting that the increase of the cytosolic fluorescence occurred as a result of EV rupture. Representative of six similar experiments.

The appearance of cytosolic diS‐Cy5 fluorescence in CCK‐stimulated cells with intact plasma membrane was also observed in experiments utilizing small pancreatic tissue sections (Fig. 4), which have not been subjected to collagenase treatment. These experiments indicate that the described phenomenon is not limited to enzymatically‐isolated acinar cells or small acinar cell clusters.

We observed that, although some EVs are fragile and undergo rupture, others are robust and can retain fluorescence probe for many hours. This apparent heterogeneity of the vacuoles suggested that the acinar cells contain a stabilizing factor protecting some but not all vacuoles and that the loss of such a factor could be the mechanism behind the vacuole fragility and rupture. F‐actin is an obvious candidate for this role, particularly considering the prominent role of F‐actin in compound exocytosis of zymogen granules (Larina *et al*. 2007).

**Figure 5:**
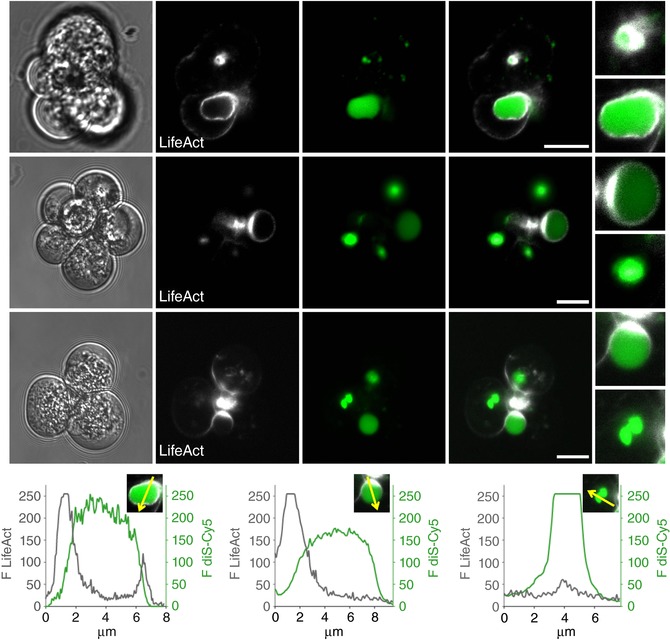
LifeAct staining reveals an association of F‐actin with EVs Pancreatic acinar cells were stimulated by CCK 10 nm for 30 min. Scale bar = 10 μm. Transmitted light images of pancreatic acinar cells are shown in the first column. The actin distribution was revealed by LifeAct expressed in the live acinar cells (second column) and correlated with the fluorescence of endocytosed diS‐Cy5 (third column). Merged images are shown in fourth column. The fifth column shows individual vacuoles on an expanded scale. The upper row of images illustrates complete coats of F‐actin on EVs. The central row shows one large vacuole with asymmetric complete F‐actin coat and a number of smaller uncoated vacuoles with little or no associated actin. The bottom row of images shows EVs with incomplete F‐actin coat and uncoated EVs with little or no associated F‐actin. The traces show examples of fluorescence intensity profiles reflecting relative localization of actin (LifeAct staining, black traces) and endocytosed diS‐Cy5 (green traces). The fluorescence intensities were measured along the arrows indicated on the images.

**Figure 6:**
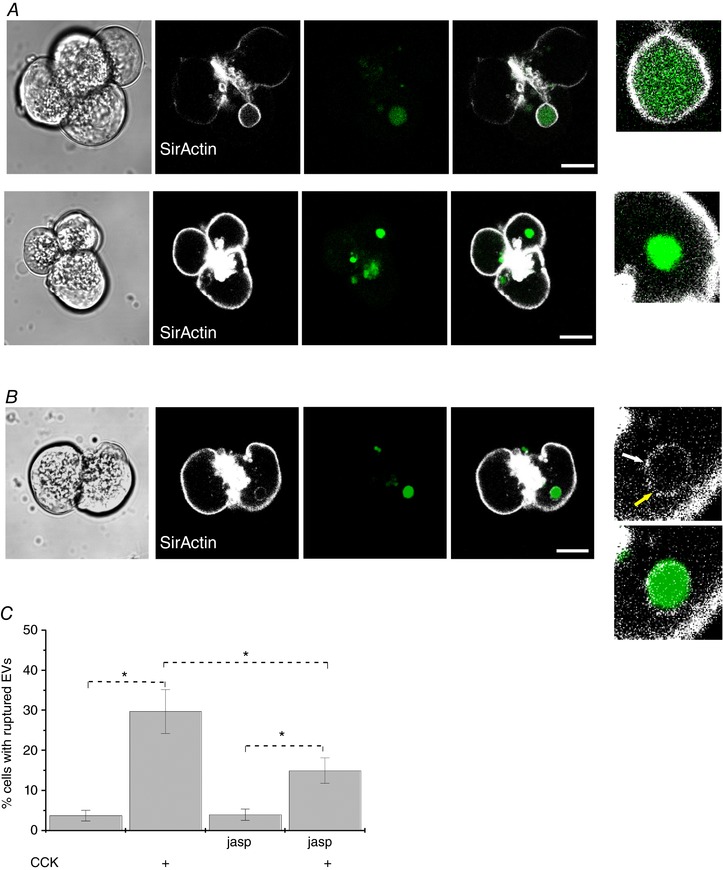
Interaction of F‐actin with EVs and the effect of Jasplakinolide *A*, actin distribution was revealed by SirActin (second column) and correlated with endocytosed probe (third column) in fixed pancreatic acinar cells. Merged images are shown in the fourth column. The fifth column shows individual vacuoles on an expanded scale. The upper row of images shows an EV coated with F‐actin. The lower row of images shows an EV with no associated F‐actin. Scale bars = 10 μm. *B*, apparent non‐uniformities and fenestrations of the actin coat. The sequence of images is similar to that described in (*A*). Scale bars = 10 μm. The fifth column illustrates actin coat and a merged image on an expanded scale. The yellow arrow shows an apparent fenestration of the actin coat, whereas the white arrow indicates an increased density. *C*, treatment of the acinar cells with 1 μm jasplakinolide (Jasp) induced a significant (*P* = 0.03) reduction in cytosolic diS‐Cy5 in CCK‐stimulated cells (*n* = 12 for all experimental conditions).

We have expressed LifeAct (a marker of F‐actin) in pancreatic acinar cells and discovered an actin coat on EVs. In cells stimulated by 10 nm CCK, ∼28% of EVs were actin‐coated (*n* = 89) (Fig. 5). In around one‐third of these vacuoles, the actin coat was incomplete or fenestrated (Fig. 5). Importantly, a significant proportion of EVs had no actin coat (72%, *n* = 89) (Fig. 4). Similar observations were made in cells stained with SiR‐actin (a membrane permeant fluorogenic probe for actin) (Lukinavicius *et al*. 2014). SiR‐actin staining revealed both actin‐coated EVs (37% of EVs, *n* = 119) (Fig. 6 *A*, upper) and uncoated EVs (63% of EVs, *n* = 119) (Fig. 6 *A*, lower). Although both actin staining methods revealed similar phenomena, the proportion of uncoated EVs was somewhat smaller for SiR‐actin. Similar to the staining with LifeAct, SiR‐actin staining revealed non‐uniformities and fenestrations of the actin coat on EVs (Fig. 6 *B*). We next investigated the putative role of actin in the rupture (or rupture prevention) of EVs using actin‐modifying compounds latrunculin‐B and jasplakinolide.

Latrunculin‐B (10 μM) application had no resolvable effect on the CCK‐induced increase in the proportion of the cells with elevated diS‐Cy5 fluorescence (*n* = 12 cell preparations in both the control group and the latrunculin‐B treated group) (not shown).

**Figure 7:**
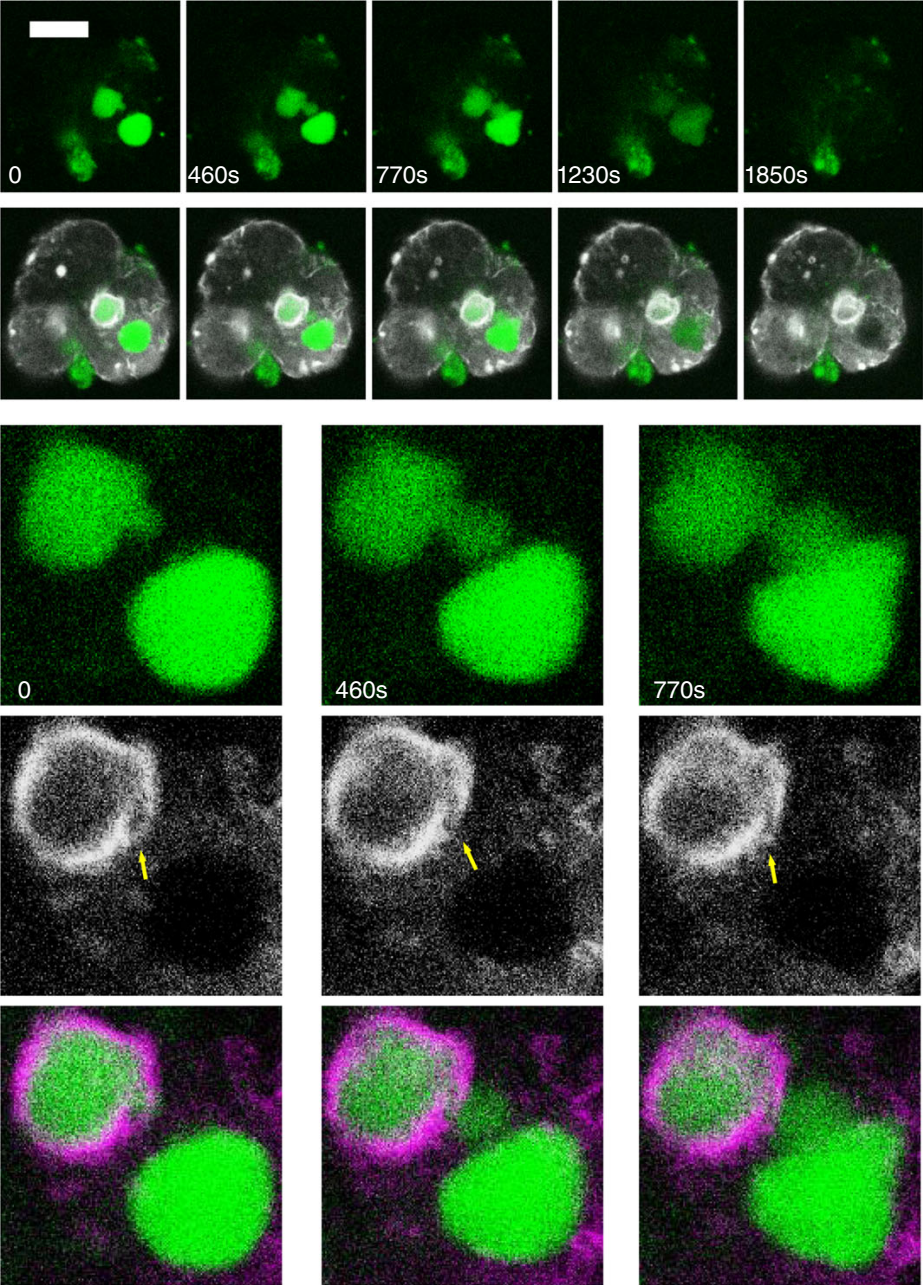
Actin staining and rupture of EVs Cells were stimulated by 10 nm CCK. Endocytosed diS‐Cy5 is shown in green. The upper gallery of images shows the ruptures of two EVs. LifeAct staining is shown in white. Scale bar = 10 μm. The lower gallery of images depicts the fragment containing the two large vacuoles undergoing rupture (corresponding to the first three images of the upper gallery). Note the fluorescence probe escaping the upper vacuole via the fenestration in the actin coat (indicated by the yellow arrow). Merged images are shown in the bottom row. In the merged images, actin is shown in magenta. Both vacuoles undergo shape changes before the rupture but these changes are particularly prominent in the lower vacuole, which is not coated with actin.

At the relatively low concentration (1 μm) used in our experiments, jasplakinolide did not change the number of EVs per cell (*n* = 112 cells in the control group and *n* = 103 cells in the jasplakinolide‐treated group) (not shown). There was no statistically significant difference in the size of the vacuoles between control (1.81 ± 0.07 μm, *n* = 223 vacuoles) and jasplakinolide‐treated (1.89 ± 0.07 μm, *n* = 239 vacuoles) groups (*P* = 0.41). However, incubation with 1 μm jasplakinolide induced a moderate but statistically significant (*P* = 0.03) decrease in the proportion of cells with elevated cytosolic diS‐Cy5 fluorescence (Fig. 6 *C*). Jasplakinolide is an actin‐stabilizing drug (Bubb *et al*. 1994) and our findings suggest that the actin surrounding EVs may play a role in reinforcing these organelles and preventing their rupture or leakage. This notion was supported by the recording of a rare phenomenon: the vacuole rupturing through the narrow fenestration in the actin coat (Fig. 7, upper vacuole).

### Exocytosis of EVs

**Figure 8:**
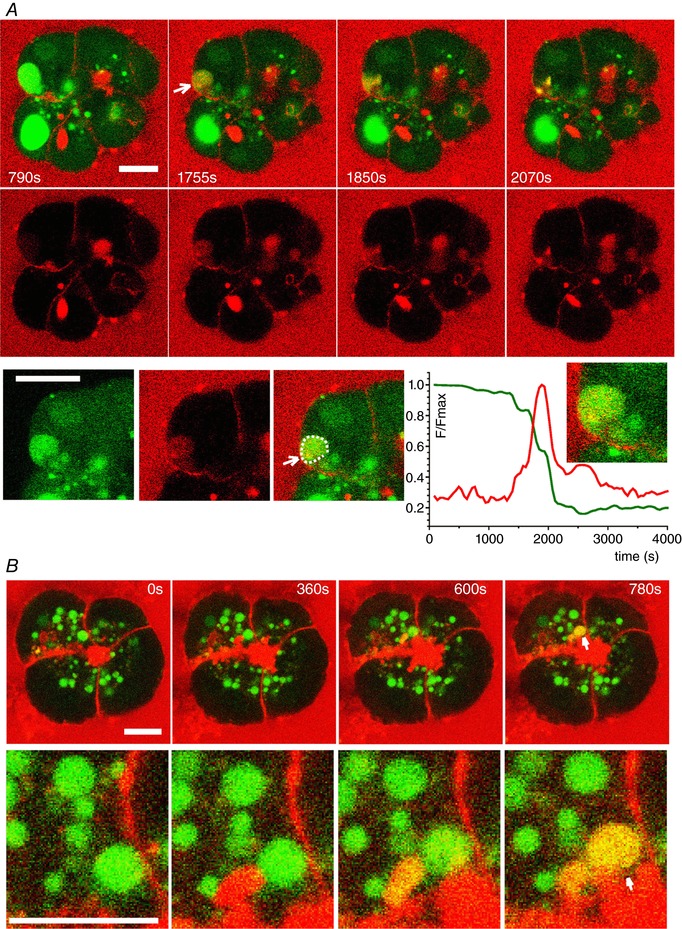
Exocytosis of EVs *A*, fusion of LY‐filled EV (green; the EV undergoing fusion is highlighted by an arrow) with the basal plasma membrane of a CCK‐stimulated acinar cell. The extracellular medium contains TRD (red) and a fusion event is characterized by mixing of the probes (yellow). Following fusion, the EV loses LY and gains TRD. The region containing the fusing EV is shown in the bottom row of images. The graph shows the time‐course of fluorescence of the two dyes recorded in the EV (highlighted by the dashed line). *B*, LY‐filled EV (green) fuses with TRD‐filled (red) post‐exocytic structure in the apical region of the CCK‐stimulated cell. The region containing the fusing EV (white arrow) is shown in the bottom row of images. Following fusion, the composite structure contains both probes (yellow) (representative of *n* = 7 observations). Scale bars = 10 μm.

During our investigation of the rupture of EVs, we serendipitously discovered another mechanism allowing trypsin to escape from the confinement of an EV; this mechanism involves the exocytosis of EVs. To systematically investigate the ability of EVs to undergo fusion with the plasma membrane, we utilized sequential incubation of the cells in two cell‐impermeable dyes: LY and TRD. The rationale of these experiments was that the exchange of fluorescent molecules between the EVs and the extracellular solution should definitively identify the fusion events. In these experiments, cells formed LY‐filled EVs during 30 min of incubation with LY in the presence of supramaximal CCK. The cells were then washed and again stimulated by supramaximal CCK in the presence of TRD. A number of new EVs filled with TRD were formed (Fig. 8 *A*). Importantly, some of the LY‐filled EVs, found in basolateral regions of the cell, were able to exchange the fluorescence probes with the extracellular solution (manifested by the loss of LY and the uptake of TRD), confirming fusion with the basolateral part of the plasma membrane (*n* = 11) (Fig. 8 *A*). As in the case of recording ruptures of EVs, the direct imaging of fusion of EVs was time consuming. A confocal observation time of ∼15.3 h (920 min) was required to resolve a single fusion event. In addition to the basolateral fusion events, some of the LY‐filled EVs fused with TRD‐filled post‐exocytic structures at the apical part of the cells, indicating that EVs can also undergo secondary exocytosis in this region (*n* = 7) (Fig. 8 *B*).

## Discussion

Pancreatic acinar cells utilize compound exocytosis to secrete zymogens. Large EVs form in pancreatic acinar cells as a result of aberrant compound endocytosis of secretory (zymogen) granules (Sherwood *et al*. 2007; Voronina *et al*. 2015). Comparinge the size of EVs with that of secretory granules, we can conclude that tens and even hundreds of secretory granules first have to form a post‐exocytic Ω‐shaped structure and, subsequently, this structure has to disconnect from the plasma membrane to form the large EVs (up to 12 μm in diameter) observed in the acinar cells stimulated with TLC‐S or supramaximal CCK. Trypsinogen activation observed in EVs probably occurs because not all zymogen is released from the post‐exocytic structures through the fusion pore (which is known to be restricted and rather short‐lived) (Larina *et al*. 2007).

The EVs containing potentially damaging digestive enzymes, notably trypsin, should be harmless until the enzymes escape into the cytosol or extracellular solution. The rupture and fusion of the EVs, as described in the present study, delineate the escape routes that probably initiate autodigestion of the organ.

Rupture of a large EV is a catastrophic event for the cell, usually resulting in cell death within 30 min. The large size of EVs, formed by the inducers of acute pancreatitis, allowed us to definitively resolve ruptures of these organelles, although experiments aimed at imaging ruptures of the EVs were time consuming and the majority of confocal imaging sessions ended without resolving a single EV rupture. However, even the substantial experimental time necessary to resolve a single rupture (8 h) is still shorter than the time required for inducing acute pancreatitis in the cerulein model (12 or 24 h) (Lerch & Gorelick, 2013). In our experiments on isolated acinar cells and cell clusters, we found that ∼40–50% of cells will have at least one large vacuole (of the size that we used to image ruptures) (Fig. 1 *C*). Considering that the frequency of vacuolar ruptures is approximately one rupture per 8 h, we can assume that ∼20–30% of cells will experience a catastrophic rupture of an EV during the time necessary to induce acute pancreatitis in an animal model. Assuming similar rates of cellular vacuolization and vacuolar rupture in the pancreata of mice undergoing induction of acute pancreatitis, it could be inferred that a significant proportion of the acinar cells of the glands (perhaps 20–30% of cells) may be damaged by the processes initiated by vacuolar rupture.

The experiments utilizing a complimentary approach for detecting the escape of the content from the vacuoles into the cytosol (relying on the distribution of cytosolic fluorescence intensities, as shown on the Fig. 3) also suggests that a substantial proportion of cells experienced ruptures or leakage of EVs following supramaximal CCK stimulation. The increases of cytosolic fluorescence recorded in these experiments are usually very small and comparable to autofluorescence of the cells in the red part of the spectrum. This technical approach does not reveal the identity of the ruptured vacuoles (e.g. some of the vacuoles could be small and incapable of inducing cell death by releasing vacuolar content), although the high proportion of affected cells (∼30%) further highlights the importance of this phenomenon.

The results of the present study suggest that intravacuolar trypsinogen activation and cathepsin B activity are not essential for the rupture of EVs. So what is the mechanism rupturing these organelles? In the present study, as well as a previous study (Sherwood *et al*. 2007), we have observed long‐distance movement of EVs and considerable changes of their shapes. A clear example of the change in the shape of a vacuole before rupture is shown in Fig. 7 (lower vacuole). These movements and shape changes probably reflect interactions of EVs vacuoles with cytoskeletal elements and/or cellular organelles in the very dynamic and crowded interior of acinar cells. We consider that the forces produced by such interactions could be responsible for rupturing EVs. In the present study, we discovered and characterized the F‐actin coating of EVs. Such actination probably serves to protect EVs from rupture and, consequently, the loss of actin coat could make the large EVs fragile and unstable. The dynamics of F‐actin on the EVs is now the subject of a separate investigation in our laboratory.

The observation time necessary to resolve a fusion event between an EV and the basolateral plasma membrane (15.3 h of confocal imaging) is substantial but similar to the induction time in some models of acute pancreatitis (Lerch & Gorelick, 2013). Therefore, one or more fusion events will probably occur during the induction time. The fusion of trypsin‐containing vacuoles with the basolateral membrane probably occurs because of the documented basolateral appearance of proteins involved in exocytosis in this cell type under pathological conditions (Dolai *et al*. 2012; 2018). Formation of EVs depends on exocytosis of zymogen granules (Sherwood *et al*. 2007). Actin plays an important role in exocytosis of zymogen granules in pancreatic acinar cells. Indeed, stabilization of actin by phalloidin or jasplakinolide inhibits this process (Muallem *et al*. 1995; Valentijn *et al*. 2000). It has been clearly documented that, in pancreatic acinar cells, zymogen granules are coated by actin (Valentijn *et al*. 2000; Nemoto *et al*. 2004; Jang *et al*. 2012), although only after they fuse with the plasma membrane (Jang *et al*. 2012). This suggests that normal exocytosis of secretory granules occurs when granules do not have an actin coat that could prevent the contact between the granule and the plasma membrane. The same consideration probably applies to the exocytosis of EVs that are derived from the secretory granules. The actin coat should therefore prevent both rupture and fusion of EVs.

The fusion of EVs with the apical part of the plasma membrane presumably utilizes the same mechanism as that used by secretory granules (Thorn & Gaisano, 2012). In this case, trypsin and other digestive enzymes could be delivered directly into the lumen of pancreatic ducts. The degree of the damage to the ducts and the organ as a whole in this scenario probably depends on the efficiency of fluid and bicarbonate secretion (Pallagi *et al*. 2011; Pallagi *et al*. 2015). Notably, trypsin reduces ductal bicarbonate secretion (Pallagi *et al*. 2011). The effect of trypsin secreted from EVs could be further amplified by other cells of the acinus delivering zymogens by ‘normal’ release from secretory granules to the same duct and subsequent activation these zymogens by trypsin released from EVs.

The present study describes an important step in the pathway initiated by inducers of acute pancreatitis. It follows aberrant Ca^2+^‐dependent compound endocytosis and the formation of large EVs. It also involves ruptures of EVs and their fusion with the plasma membrane, comprising processes that mediate the release of trypsin into intracellular and extracellular milieu.

## Additional information

### Competing interests

The authors declare that they have no competing interests.

### Author contributions

MC, FDF, DM, MWS, MA and SV contributed to the experimental part of the project. SV, DNC, RS, LH and AVT designed and supervised the project. All authors approved the final version of the manuscript and agree to be accountable for all aspects of the work in ensuring that questions related to the accuracy or integrity of any part of the work are appropriately investigated and resolved. All persons designated as authors qualify for authorship, and all those who qualify for authorship are listed.

### Funding

The study was supported by a Medical Research Council (UK) grant (MR/K012967/1) and by a Wellcome Trust grant (105273/Z/14/A).

**Translational perspective**

The present study shows that the endocytic vacuoles, as formed in pancreatic acinar cells in pathological settings, may rupture and precipitate acinar cell death. Endocytic vacuoles contain zymogens, including trypsinogen, which can undergo activation inside these organelles. Although this activation does not affect the rupture process itself, it may be critical for the downstream events and cell fate. On the other hand, the aberrant endocytosis followed by the rupture of endocytic vacuoles delineates a potentially novel route for the delivery of membrane impermeant molecules to the cytosol of the acinar cell. It is conceivable that, in the future, this pathway could be utilized to deliver pharmacologically relevant compounds with the aim of minimizing or preventing acinar cell damage. Notably, such membrane‐impermeant compounds will reach the cytosolic targets only in vulnerable acinar cells that have experienced the sequence of aberrant endocytosis and rupture of the endocytic vacuoles. The present study also highlights the possibility of the re‐uptake of zymogens (and potentially active proteases) into endocytic vacuoles from the acinar lumen. This is a novel route for the delivery of damaging enzymes into endocytic structures and later to the cellular cytosol. The existence of this route further highlights the importance of an efficient acinar and ductal fluid secretion as an endogenous mechanism for the prevention of acute pancreatitis and emphasizes the potential utility of developing treatments that normalize or stimulate fluid secretion.
